# What are people’s experiences of orthorexia nervosa? A qualitative study of online blogs

**DOI:** 10.1007/s40519-019-00809-2

**Published:** 2019-11-13

**Authors:** Maddy Greville-Harris, Janet Smithson, Anke Karl

**Affiliations:** 1 Bournemouth University, Poole, UK; 2 University of Exeter, Devon, UK

**Keywords:** Orthorexia nervosa, Clean eating, Eating disorder, Qualitative research, Thematic analysis, Blogs

## Abstract

**Purpose:**

Orthorexia nervosa (ON) is a proposed new eating disorder, used to describe a pathological obsession with healthy or ‘clean’ eating. Although some quantitative research has been carried out in ON, very little qualitative work has been published to date to explore individual experiences of ON. Thus, this study aimed to explore individuals’ personal experiences of ON, as described in online blogs.

**Methods:**

Fifteen women bloggers, who self-identified as having ON, consented for their blog entries to be analysed in this study. Forty pre-existing blog entries describing the first-person experiences of ON were analysed using thematic analysis.

**Results:**

Three key themes were discussed: (1) initial motivations for a healthier lifestyle, (2) fuelling the problem—social influences, and: (3) when healthy becomes unhealthy. Bloggers described the role of social messages, comparison with others around ideas of ‘healthiness’, as well as confusion around diagnosis as factors influencing their disordered eating. They also described the exacerbating impact of perfectionism and perceived control, as well as a confirmatory cycle of fear and avoidance. For some bloggers, increased physical symptoms in response to feared foods provided confirmation for these fears, further exacerbating food avoidance.

**Conclusion:**

Whilst the debate around the diagnosis of ON continues, these bloggers’ accounts suggest that ON is experienced as a legitimate, debilitating disorder, worthy of clinical and research investigation. This study provides evidence of some of the potential triggers and maintaining factors for this disordered eating style.

**Level of evidence:**

Level V, qualitative descriptive study.

## Introduction

Societal attitudes towards healthy eating are changing, with an increasing emphasis on eating good quality or ‘clean’ foods. A preoccupation with healthy eating is idealised in our society [1], with the focus on what, when, and how much, to eat becoming an important part of social discourse [2]. Thus, ‘clean eating’, or the strict avoidance of foods considered ‘impure’ or ‘unhealthy’, is an increasingly popular, and arguably dangerous, dietary trend [3]. Perhaps due to this emphasis on avoiding ‘unhealthy’ foods, a potentially new eating disorder, orthorexia nervosa (ON) has come to light.

ON describes an obsession with ‘clean’ eating which becomes extreme and pathological, resulting in health problems and/or impairment to functioning [4]. Several researchers have suggested diagnostic criteria for ON [5–7], yet there is still no consensus. However, what these authors agree upon, is that ON involves: (1) obsessional preoccupation with ‘healthy’, ‘pure’, or ‘clean’ foods, (2) rigid avoidance of foods considered ‘unhealthy’ or ‘unclean’, (3) distress at violation of food rules, and (4) impairment to social, physical, and/or psychological wellbeing resulting from these food beliefs and behaviours.

There is plenty that is not yet known about the development and maintenance of ON, as well as whether it warrants recognition as a distinct eating disorder (ED) classification. ON is suggested to be distinct from Anorexia Nervosa (AN), in that it focuses upon a pathological obsession with the quality, rather than the quantity of food [6]. However, ON is thought to share common traits with AN, such as perfectionism and a high need to exert control [8]. Indeed, there is debate as to whether ON could serve as a phase within an established ED, such as AN. ON has been suggested as a risk factor for developing an established ED [9] and as a ‘healthier way’ to control food during ED recovery [10]. Given the current interest in health food diets, Segura-Garcia and colleagues [10] also suggest that ON may serve as a more socially acceptable method of restricting foods, and thus could co-exist with other established eating disorders.

While some researchers have proposed that ON may be a subtype of AN, others propose that ON may not fit into an ED classification at all, instead representing a subtype of obsessive compulsive disorder (OCD) [8]. Whilst ON has been suggested to be similar to OCD in terms of the recurrent intrusive nature of thoughts about food, increased concern over contamination of food, and ritualised patterns of eating [8]. Koven and Abry [8] suggest that the nature of the obsessions is very different. Whereas OCD obsessions tend to be ego-dystonic, ON obsessions are ego-syntonic (i.e., in line with the individual’s sense of self). Indeed, it is suggested that ON is characterised by feelings of superiority over individuals who eat foods considered to be ‘unhealthy’ [4].

Although not yet recognised as a distinct ED by the International Classification of Diseases [11] or Diagnostic and Statistical Manual of Mental Disorders [12], ON has been recognised by clinicians as an area necessitating more research [13]. For example, Vandereycken [14] collected survey data from 111 Dutch-speaking ED professionals. About two-thirds of respondents reported that they had observed ON in their practice and believed that ON deserved attention both in research and clinically.

Existing research examining ON has been limited, with a recent systematic review [15], concluding that ON research so far is “disparate” (p. 12). Cena and colleagues’ narrative review of the research to date [16] also outlines the lack of consensus in the literature in terms of definition and proposed diagnostic criteria for ON. Moreover, Dunn and Bratman’s recent review [6] concluded that there are currently shortcomings in the measurement tools and prevalence ratings for ON to date. Nevertheless research looking at ON is rapidly expanding; Missbach and Barthels [17] found 47 published articles on ON in PubMed, 70% of which were published within the last 5 years. However, Costa, Hardan-Khalil, and Gibbs [18] conclude that “there is still little original research establishing diagnostic criteria, clear symptomology, and effective treatment modalities for ON” (p. 987).

Despite the lack of empirical literature to date, ON is an important topic, with many media and clinical cases coming to light. Media interest began in 2014, when a prolific vegan blog writer admitted to her 70,000 Instagram followers that she had an ED based on the quality of her food intake [6]. Since then, blogs and forums discussing the construct of ON, as well as people’s experiences of the disorder, have emerged. These online sources provide a rich source of data about people’s subjective experiences, which have so far mainly been ignored by ON researchers.

First-person qualitative accounts are potentially useful for ON research. Haman and colleagues [15] state that there is a gap in the existing ON literature due to the lack of qualitative studies in this area. He proposes that qualitative research is necessary to explore individuals’ experiences of ON, as well as to “emphasize a bottom-up perspective and ensure that the voices of individuals are heard” (p. 13). To our knowledge, there is only one qualitative study to date which explores individual accounts of ON [19]. However, this work does not explore potential factors leading to the development and maintenance of ON from a clinical perspective, seeking to understand these experiences in depth.

This study seeks to address this gap, by exploring the personal experiences of ON as described in pre-existing online blogs. Online blogs refer to “posts on a common web page, usually written by a single author” (p. 92) [20]. Blogs provide useful material for qualitative analysis, because they are publicly available naturalistic data, allowing access to populations that may be socially or geographically separated from the researcher. This study thus aims to answer the following research question: what are people’s personal experiences of ON, as described in online blogs?

## Methods

### Data collection

Pre-existing online blogs describing personal experiences of ON during adulthood were the focus of this study. Blogs were included if: (1) the blogger was aged 18 or over, (2) the blog described the blogger’s personal experiences of ON, (3) the blog was written in English, and (4) the blogger used the keyword “orthorexia”. Blogs were excluded if the blog described ON from an outsider/expert perspective (without describing personal experiences of ON), or the keyword “orthorexia” was not used by the blogger to describe their experiences.

### Procedure

Initially, feedback was sought from two individuals with lived experience of ED who used Internet sites and blogs, recruited through the B-EAT ED charity. These individuals provided feedback on the study documents and were provided with remuneration for their time and input.

To identify blogs for this research, “orthorexia blog”, “my orthorexia story”, and “my orthorexia journey” were entered into Google search engine. Blogs retrieved over the first ten pages of search results were viewed for suitability. Bloggers whose blogs met study eligibility criteria were contacted online. These bloggers were provided with an information sheet and a link to an online consent form. Thirty-one eligible blogs were identified and attempts were made to contact their blog authors. Three bloggers did not provide contact details, two declined to take part, one expressed interest, but did not give consent, and ten did not respond. The remaining 15 eligible bloggers consented to include their blog entries in this study.

Identified blogs were searched with the keywords “orthorexia” and “orthorexic” to ensure that all relevant blog posts within each blog were included. One blogger consented to the inclusion of her supplementary video blog (‘vlog’), which was transcribed and added to the data corpus. Forty relevant blog posts were thus included from the 15 consenting bloggers. Included posts were copied and pasted into a Word document, retaining any graphics, emoticons, punctuation or grammatical/spelling errors. Data were then imported into NVivo 10 software (QSR International, 2012) for analysis.

### Ethical considerations

Ethical approval was obtained from the University of Exeter Psychology Ethics Board (Project 2017/1509). Participants were informed of their right to withdraw from the study during the data collection period and were provided with a debriefing form once the study had finished.

The British Psychological Society [21] guidelines for Internet-mediated research were consulted during study design, particularly when considering confidentiality. In line with other researchers [22, 23], it was initially decided that bloggers’ identities should be protected by removing all names and identifying information from the data, However, one blogger stated that she would like her blog material to be cited and identifiable. Thus, an amendment to ethical approval was obtained to allow participants the choice to remain anonymous or be cited in the write-up. Twelve bloggers stated that they were happy to be cited and named in this study, one blogger asked to remain anonymous, and two did not respond (and thus were anonymised). For bloggers who were included anonymously, quotes used in the write-up were not reported verbatim, to ensure that they could not be traced back to their original source [23].

### Analysis

Data were analysed using thematic analysis based on Braun and Clarke’s framework [24]. Thematic analysis is theoretically flexible [24], and allows for meaningful patterns to be explored within the data [25]. Braun and Clarke [24] provide comprehensive guidelines on the stages of thematic analysis [24] which were used here: (1) familiarisation with the data, (2) generating a list of initial codes, (3) collating codes into themes and reviewing these themes, (4) defining and labelling themes, and: (5) selecting illustrative quotes and linking findings back to existing literature.

To ensure that data analysis was coherent and transparent [26], a data trail was kept, using concept cards [27]. Deviant case analysis allowed for contradictory positions and voices to be explored [28]. A second coder also coded four transcripts to ensure consistency in identified themes. Similar codes were identified by both coders, and any discrepancies were discussed and resolved.

## Results

### Participant demographics

All the recruited bloggers were female, aged 19–32 years. Twelve bloggers were from the USA, with three bloggers identifying as from England, Australia, and India, respectively. Table 1 outlines the named bloggers and the number of blog posts per participant.

**Table 1:** Demographic information for participants

Source	No. of included blog posts
Anon1	3
Anon2	2
Anon3	2 (including 1 vlog)
addictedtolovely.com	1
autumnbrianne.com	4
Ashley Bailey, http://108squaremiles.com/	5
eatingrules.com/orthorexia/	1
emilyfonnesbeck.com	6
geekie-chic.blogspot.co.uk	1
http://thepurplefig.com/calorie-counting-kept-me-isolated-a-story-about-orthorexia/	1
experiencelife.com/author/kdalebout/	1
maddymoon.com	5
nourisheveryday.com	2
heathercaplan.com/realtalk/	5
winetoweightlifting.com/	1

### Overview of themes

Three main themes comprising seven subthemes were identified (see Table 2).

**Table 2:** Themes and subthemes identified in this analysis

Theme	Subthemes
Initial motivations for a healthier lifestyle	Quest to find what is wrong Desire to do right
Fuelling the problem—social influences	Unhelpful health claims Comparing ‘healthiness’ Confusion around diagnosis
When healthy becomes unhealthy…	Punishing drive for perfection and control A confirmatory cycle of fear and avoidance

### Initial motivations for a healthier lifestyle

The bloggers described how their desire to do right when it came to health and food, and/or their quest to cure their health difficulties, were strong motivators for making initial dietary changes.

#### Quest to find what is wrong

Four bloggers described how initial health problems (often digestive issues) led to a quest to find what was wrong with their bodies and discover what needed to be ‘fixed’. This led to eliminating certain food groups.

“I have been on an endless search to heal various physical ailments through food elimination and diets… During this same time, I continued to suffer from gastrointestinal issues … So, in my mind, there was still something wrong. There was still something to fix…. The search consumed me.” (Ashley Bailey)

The initial health benefits experienced by themselves or their families spurred them on with their diets. However, these initial benefits were not always long-lasting, the dietary cuts were sometimes difficult to reverse, and so the quest to “fix” their problem continued.

#### Desire to do right

The start of ON for many bloggers, was a decision to be healthier, which spiralled into disordered eating. Some described how this initially involved exercising more, calorie counting, or adhering to specific diets such as paleo or plant-based.

“And so my “health transformation” began. I went from Friday night regulars at Hungry Jacks to making absolutely all my own “healthy food”, and I started running and doing as many gym classes as I could squeeze in. … Yep, I’ve got this, I thought.” (Nourish Everyday)

Diet was used to strive for ‘health’ and in some cases for ethical reasons or weight loss. However, ideas around ‘health’ were often individual and idiosyncratic, shifting over time.

“When I gained a bit of weight as a college freshman, I began counting calories and adjusted my diet. I transitioned from vegetarian to vegan…After that, I went gluten-free to further “perfect” my diet. Then I decided I’d eat nothing but raw foods, and I quit carbs and sugar, too. Eventually, the only thing left was raw vegetables…” (Experience Life)

The confusion around what constitutes ‘healthy eating’ caused some bloggers to transition through more extreme elimination diets, in line with their changing beliefs around ‘healthy/unhealthy’ and ‘clean/unclean’ foods.

### Fuelling the problem—social influences

Social influences, such as unhelpful health claims and social comparison, were described by many as promoting their disordered eating. Some described how whilst they appeared healthy to others, their underlying difficulties with food were hidden. Their difficulties were further compounded by the confusion around diagnosis and lack of recognition of ON.

#### Unhelpful health claims

Many bloggers explained how the Internet, social media, and magazines normalised the notion that detoxing, restricting, and eliminating foods was healthy, fuelling their disordered eating.

“Orthorexia runs rampant on “healthy eating” blogs and Instagram accounts, in cleansing or detoxing programs, and with nutrition “experts” claiming you MUST cut X, Y, and Z out of your diet. All of the above have normalised this fixation on idealizing certain types of food.” (Real Talk)

Bloggers often believed and invested in these fear-based messages, researching how to better achieve ‘health’, often searching online or in magazines.

“I bought in heavily to the low carb, low fat mantra that dominated popular media, shrinking my diet to controlled portions… I was scouring every women’s mag going, flipping to the diet and lifestyle section and cycling through all of the 1,200–1,500 cal-per-day meal plans I’d find in there for more ideas.” (Nourish Everyday)

The many contradictory, and sometimes extreme, social messages about what constitutes ‘health’ and ‘unhealth’, thus shaped the diets that many bloggers invested in, in their desire to be healthy, and/or to ‘fix’ their health problems.

#### Comparing ‘healthiness’

Bloggers compared diets, appearance, and lifestyle with others, for example with friends, magazine images, or on social media. Some stated that upward comparisons about health or weight (i.e., comparison with those perceived as following a ‘healthier’ lifestyle) left them feeling below standard, leading them to instigate more food rules.

“I developed ideas about “okay” and “bad” foods based on people I admired. It wasn’t just one person or account, but a combination. I believed that if I ate what they ate, I would end up looking like them…” (Anon)

Some described how downward comparisons (i.e., with those perceived as less ‘healthy’) about health and calorie consumption led them to make judgements about other’s lifestyle choices, bringing a sense of superiority, that they were doing ‘better’ at being healthy than others.

“I became super judgmental of anyone that wasn’t eating paleo. In fact, I even became judgmental of those who were eating paleo. …. where are your organ meats and fermented foods? Why aren’t you brewing bone broth and drinking kombucha? I was not perfect in everything that I was doing, but boy, was it easy to point out that everyone else was doing it wrong.” (Wine to Weightlifting)

Many bloggers started their own nutrition blogs, shared diet/health pictures on Instagram, or worked professionally advising others on healthy eating. Bloggers stated that things were “not as they appeared”. Whilst receiving praise and admiration for their apparently ‘healthy’ lifestyle, their underlying difficulties often remained hidden unless their weight loss was extreme.

“I think to an outsider I looked really healthy, fit, caring about my diet… but mentally I worried about what I ate constantly”. (Anon)

Three bloggers described the realisation that, they too, began to proliferate unhealthy messages about health and diet that had first influenced them, and thus, the cycle of fear-based food messages continued.

#### Confusion around diagnosis

Bloggers described the implications of the widespread lack of awareness and official diagnostic criteria for ON. The lack of recognition of ON resulted in confusion around, and delegitimization of their disordered eating.

“For a long time I considered it a mild brush with disordered eating—just a little too “healthy,” with good intentions—not something worth sharing. I thought that since I wasn’t anorexic or bulimic, it wasn’t serious.” (Real Talk)

Five bloggers described experiences of other mental health difficulties such as depression or anxiety. Although several bloggers discussed the overlap between ON and other eating disorders, one blogger explicitly stated that she did not have a history of eating disorders or issues with her body image.

“I **never** had any eating disorders before, never had any sort of self-body-hate, nor did I ever strive to look like someone in a magazine. I was naturally thin and was not switching up my diet just to look good.” (Wine to Weightlifting).

In contrast, five bloggers discussed their experiences of an overlap between ON and other EDs, particularly anorexia.

“At first, I was “just” anorexic, but as my compulsion grew, I quickly transitioned to orthorexia.” (Experience Life).

While three bloggers described transitioning from AN to ON over time, one blogger described how she felt that her ON was transitioning to AN. Thus, experiences of ON were not always clear-cut.

### When healthy eating becomes unhealthy…

Bloggers identified how their disordered eating became problematic, because it fuelled a punishing drive for perfection and control, and led to a confirmatory cycle of fear and avoidance which maintained, and further exacerbated, their disordered eating.

#### Punishing drive for perfection and control

Some stated that the need to be ‘perfect’ and ‘in control’ drove them to strive obsessively for increasingly unrelenting and unrealistic standards, until their diet and exercise regimes were extreme and debilitating.

“After a few months though—being a Type A, all-or-nothing perfectionist kind of girl—one or two lifestyle “improvements” weren’t enough…. Sweaty, extensive exercise, plus walking, was scheduled in every day, without fail. I needed to do it.” (Nourish everyday)

Rules around food and exercise were used to feel in control, as a coping mechanism to feel safe, particularly when other areas of life felt uncontrollable. Bloggers explained that the target of control could change, and thus, dietary rules might appear arbitrary, but were constant in serving the same purpose, providing a sense of perceived control for the individual.

“I didn’t know that I would replace counting calories with a low-fat obsession, which I later replaced with a variety of food aversions that transferred the sense of control from one thing to another.” (Real Talk)

Some described their later realisation that their adherence to rules had brought a ‘false sense of control’ as their obsession with control in fact ended up controlling them. For many, the unrelenting standards and critical self-talk resulted in a punishing relationship with themselves and their bodies.

“I did not know what my “natural” body looked like anymore, because I had manipulated my body for so long. I had abused her. Talked ugly to her. Worked her past her limits. Starved her. Punished her.” (Autumn Brianne)

Eight bloggers described how they engaged in restriction and deprivation, paradoxically to the detriment of health and wellbeing. Serious physical consequences included extreme weight loss, health complaints, difficulty concentrating, exhaustion, vitamin deficiencies, and amenorrhea.

#### A confirmatory cycle of fear and avoidance

Bloggers expressed an intense fear about eating certain foods and ingredients, as well as fearing certain food rituals (such as food combining) which were considered toxic or dangerous. Three bloggers stated that heightened fear of certain foods increased their physical symptoms, and caused their body to reject these foods.

“I started restricting myself to certain food items only, not because I wanted to be healthier, but because I genuinely believed that those foods would cause me physical and mental harm. I tried to give up eating cheese, butter and milk, and the more I avoided those foods, the more my body would start to reject them.” (Geekie Chic)

These bloggers believed that their digestive problems were due to physiological responses to anxiety (‘fight or flight’), with increased conviction that certain foods were a threat, exacerbating their physical symptoms.

“Do you know what physically happens to animal (including humans) when they are in ‘fight or flight’ mode?… I can’t even begin to tell you how much this resonates with me…. I’d like to especially point out the one on digestion and immune system shutting down to allow more energy for emergency functions. Wow, did it ever. Chronic diarrhoea and various other immune related symptoms is the main thing that plagued me during this time.” (Ashley Bailey)

This led to vicious cycle-anxieties around eating certain foods resulted in increased physical symptoms, thus confirming the threatening nature of these foods.

“… I have a theory … that when we consistently think negative and fearful thoughts about food, our body begins to see it as a threat. Literally. Our immune system encodes it, our digestive tract rejects it and our brains see it as a source of anxiety. What was once necessary is now the enemy - physically and psychologically.” (Emily Fonnesbeck)

Thus, fear and anxiety around ‘unhealthy’ foods, exacerbated by the societal fear-based messages, were believed by some to lead to food avoidance and increased physical health problems. Thus, health issues were fuelled by the fear itself, rather than the ‘problem’ foods per se.

## Discussion

This study addresses a significant gap in research by exploring individuals’ experiences of ON, particularly focusing upon factors described in the development and maintenance of ON. Three key themes were described: (1) initial motivations for a healthier lifestyle, (2) fuelling the problem—social influences, and: (3) when healthy becomes unhealthy. To our knowledge, this is the first qualitative study to focus on this area. The findings thus take steps in helping to understand how individuals make sense of the potential factors influencing the development and maintenance of their disordered eating in the context of ON.

First, this study is novel in identifying the potential impact of confusion around ON diagnosis for those with disordered eating. Bloggers described lack of awareness and official diagnosis of ON, causing confusion, and sometimes delegitimization, of their experiences. These narratives mirror the uncertainty around diagnostic criteria for ON in the literature, the lack of recognised diagnosis within the DSM-5 [12], and perhaps the lack of awareness of ON in the general population. Although no studies have examined awareness of ON in the UK or USA, preliminary research in Poland suggested that ON was largely unrecognised, with 71% of young people surveyed (*n* = 981) reporting that they did not know what orthorexia was [13].

Second, this study highlights the possible trajectories into ON and the potential crossover with other EDs. Indeed, whether there is diagnostic crossover between ON and other EDs, and the nature of this relationship, is a source of some debate. For example, whilst the proposed motivation for ON is stated as health rather than weight (i.e., the quality rather than quantity of food), Bratman [29] acknowledges that this distinction can be problematic, as notions of ‘health’ can come to incorporate ideas about weight and weight loss. Bratman suggests that the concepts of “losing weight, improving health and enhancing healthy appearance” have become melded (p. 384), thus bringing ON and AN closer together. The ON literature suggests that ON could be a gateway into (or out of) another recognised ED. ON is proposed as a ‘healthier’ means to control food during ED recovery [8] as well as a risk factor for developing a more ‘severe’ ED [9]. Whilst more research is needed to understand these different ED trajectories, in this study, ON was not described as merely a recovery stage from another ED, but rather as a difficulty that was severe, distinct, and debilitating enough to be investigated, both in relation to other disorders, and in its own right.

A third important finding for this study was in highlighting the importance of the current social climate in fuelling and maintaining ON. Bloggers described how social messages about health and diet from the Internet, social media, and magazines normalised fear-based ideas about food, and encouraged the notion that detoxing, restricting, and eliminating foods was the healthiest choice. For these bloggers, it may be that the internalisation of online fear-based and idealised messages about diet promoted their orthorexic behaviours. The relationship between social media and EDs more generally is widely established [30]. Yet, much of this work has focused on the influence of thin ideals on EDs [31–34]. However, the experiences of many of these bloggers suggested that the focus of ON was on ideals around health and avoiding illness, rather than thinness per se. Whilst this focus is in line with the proposed diagnostic criteria for ON [5–7], it differs from other established EDs, and warrants further investigation.

As well as social messages about health, bloggers in this research reflect on the hidden nature and social acceptability of their ON. These women described how their disordered eating remained unnoticed by many unless weight loss was extreme. In addition, many described receiving praise and admiration for their lifestyle. Some admitted to an outward appearance of health and wellbeing, when in reality, “things were not as they appeared”. ON has been postulated to be an ego-syntonic disorder [8], resulting in a sense of superiority for the individual, intolerance of other’s food beliefs, or the flaunting of one’s own habits [35, 36]. In addition, ON has been described as a “disease disguised as a virtue” (p. 2) [37] as an obsession with the promoted idea of ‘healthy eating’.

Social comparison was described as an important influence for these bloggers, which is in line with the existing ED literature. According to social comparison theory [38, 39], individuals look for standards against which to make comparisons to assess how well they are doing. This includes making comparisons with available others, and adjusting behaviour to reduce perceived discrepancies between self and others (if the discrepancy is believed to be important). Corning, Krumm and Smitham [39] found that an increased tendency to carry out social comparisons predicted the presence of ED symptoms. More specifically, comparison of one’s body with images of other women which resulted in self-defeating appraisals predicted the presence of ED symptoms.

Whilst more research is needed to ascertain whether similar processes occur for individuals with ON in terms of health-related, rather than weight-related comparisons, our findings suggest that such processes may be important. Moreover, although existing research has focused on upward comparisons, our findings suggest that downward comparison, with those considered less ‘healthy’, may also play a role for individuals with ON in increasing self-esteem. These contrasting narratives of downward comparison and social acceptability may be particularly important in understanding how ON can go unnoticed, remain hidden, or indeed be promoted as something to aspire to.

Finally, this study illustrates possible key factors in the development and maintenance of ON, most notably perceived control, perfectionism, and a confirmatory cycle of fear and avoidance. Whilst the importance of perfectionism [40–42] and perceived control [43–45] have been highlighted for EDs more generally, little research has examined these factors in relation to ON. Barnes and Caltabiano’s survey study [46] found that higher self-reported perfectionism was significantly correlated with higher levels of reported orthorexic symptoms; however, the nature of this relationship has not been explored in detail. This study thus highlights that these factors warrant further exploration in the context of ON.

Perhaps most notably, this study highlights the importance of the confirmatory cycle of fear and avoidance in the context of ON. Some participants described how their anxieties around eating certain foods resulted in increased physical symptoms, thus confirming the threatening nature of these foods. Fear and avoidance are described as a part of Barthels et al.’s proposed diagnostic criteria for ON [7]. Moreover, the role of fear and avoidance of foods is recognised in the development of other EDs such as AN [47]. However, the physical symptoms resulting from eating feared foods, and the confirmatory nature of these symptoms in verifying the food as ‘dangerous’ have not been explored in the ON literature to date. This may be a key maintaining factor for disordered eating in ON and therefore deserves further investigation.

### Strengths and limitations

This research was completed during my training as a Clinical Psychologist. Thus, my knowledge of different models of formulation, particularly the “Five P’s model” [48], undoubtedly influenced the way in which I initially tried to understand each blogger’s experience and organise my data codes. Moreover, the lack of empirical evidence in the field of ON allowed the initial focus of this analysis to be grounded within the participants’ experiences, rather than focused on a theory.

This study prioritised the subjective experiences of individuals who identify with the label of ON, allowing a better understanding of this construct from first-person accounts. To date, very little qualitative research has been published in this area [18], and thus, a criticism of ON research is its reliance upon self-report questionnaires with limited validity [46]. This research therefore allowed for a richer exploration of people’s experiences, using bloggers’ pre-existing accounts to explore their own narratives.

However, the blogs identified in this research were written by women only, many of whom worked in roles to promote health and wellbeing (such as dieticians and life coaches). The decision to use blogs captures the experience of those willing to share their experiences in the public domain, and thus, those who do not use the Internet in this way remain unheard. Moreover, due to the use of convenience sampling, the voices of those who did not want to share their blogs, as well as the voices of individuals who experience ON from within less health-oriented professions, and those of men, are not included in this sample.

Moreover, bloggers in this sample were recruited if they self-identified as having experienced ON, yet there is still confusion around what ON is, and how it differs from other EDs. Thus, it is difficult to tell: (1) whether these participants would fit existing criteria for ON, (2) the severity and these bloggers’ disordered eating, and: (3) the nature of comorbidity with other EDs/mental health difficulties. In addition, it is unclear what motivated these bloggers to write their accounts, and thus, their narratives will inevitably be influenced by factors which shape self-report, particularly when writing for the public domain. For example, bloggers may shape their accounts to convey themselves in a certain way, may emphasise or de-emphasise certain experiences or symptoms, and may selectively recall information due to hindsight bias. These factors may shape the nature of the accounts given in online blogs.

### Recommendations for future research

Overall, these bloggers’ accounts highlight many potential areas for future research, including: (1) the link between social media use and ON, (2) the relationship between perceived control, perfectionism and ON, and: (3) the potential for physical symptoms to exacerbate food-based fears in ON. Future research exploring the accounts of males with ON, as well as the perspectives of those seeking treatment in clinical settings would be helpful to explore possible ED diagnostic crossovers in more detail. Research would also benefit from the development of accepted criteria for describing ON and focusing upon participants’ past and present ED status using validated measures. Studies situated within an ED setting may be useful to explore the clinical severity of ON, and the potential diagnostic crossovers with other EDs.

## Conclusion

This study used thematic analysis to explore bloggers’ experiences of ON, focusing on issues around diagnosis, social context, motivations for pursuing a healthy lifestyle, and the role of fear, perfectionism and control. Perhaps, most notably, some participants describe a confirmatory cycle of fear and avoidance, whereby foods, once feared and avoided, began to trigger more physical symptoms, further exacerbating beliefs about the dangers of those foods. This confirmatory cycle maybe an important maintenance factor for some individuals with ON and thus warrants further investigation. Moreover, this research suggests that whilst it is unclear whether ON is indeed a distinct condition, for some bloggers their difficulties occurred without adherence to thin ideals, or history of other EDs. This suggests that ON is indeed worthy of more clinical investigation. Whilst the debate around the diagnosis of ON continues, these bloggers’ accounts suggest that ON is experienced as a legitimate and separate disorder worthy of attention in research and beyond.
